# 

**Published:** 1894-12-01

**Authors:** 


### CONTENTS.

**The Medical Inspection of Schools** - 145
**The Early Diagnosis of Cancer of the Rectum** - 146
**Therapeutics and the Lesser Circulation.** By Sir B. W. RICHARDSON, M.D., F.R.S.—I. Preliminary Considerations - 147
**An Episcopal Surgeon** - 148
**Medical Progress and Hospital Clinics:**
"Some Causes of Febrile Disturbance in Women During the Lying-in Period" - 151
Traumatic Ulnar Nerve Paralysis.—I. - 152
PROGRESS IN SURGERY.—Surgery of the Liver and Gall Bladder - 153
Diseases of the Nervous System - 154
TOXICOLOGICAL MEMORANDA - 156
NEW APPLIANCES AND THINGS MEDICAL - 156
**Editor's Letter-Box** - 148
**Annotations.**—The Treatment of Boils—The Great Northern Central Hospital—The Possibilities of Cerebral Surgery - 149
**Notes and News** - 150
**The Institutional Workshop:**
HOSPITAL CONSTRUCTION - 157
PRACTICAL DEPARTMENTS - 158
PRACTICAL POINTS - 159
HOSPITAL MEETINGS - 159
CONSTRUCTION NOTES - 159
AROUND THE HOSPITALS - 160
**Scraps and Gleanings** - 160
**The Book World of Medicine and Science** - 161

**EXTRA SUPPLEMENT.—THE NURSING MIRROR.**

**News from the Nursing World.**—Feasts and Festivals—Charing Cross Hospital—At Windsor—Fancy versus Plain Work—Our Christmas Competition—Nursing Sisters in the Army—Nurses in East London—Nurses at Swansea—Progress in Limerick—Queen's Nurses at Inverness—Amongst the Fishermen—Short Items - lx

**Antiseptics.** By F. R. HUMPHREYS, L.R.C.P., &c. - lvii

**Where to Go** - lvii

**Presentations** - lvii

**Asylum News and Nursing** - lviii

**The American Woman at Home** III.—Her Amusements - lix

**Orphans in Cages** - lx

**Nursing in Ireland** - lx

**Wants and Workers** - lx

**Old Time Nurses.** IV.—Long Service - lxi

**Nursing on the Labrador** - lxii

**Should Fever Nurses be Trained?** - lxii

**Workhouse Infirmary Nursing Association** - lxii

